# Autologous Bone Marrow Mononuclear Cells (BMMCs) for the Treatment of Uncomplicated Grade 2 Ununited Anconeal Process (UAP) in Six Dogs: Preliminary Results

**DOI:** 10.3390/vetsci8100214

**Published:** 2021-09-30

**Authors:** Alberto Maria Crovace, Luca Lacitignola, Mariasevera Di Comite, Cosimo Esposito, Alessandro Guarracino, Edda Francioso, Francesco Staffieri, Antonio Crovace

**Affiliations:** 1 Dipartimento di Scienze Mediche di Base, Neuroscienze e Organi di Senso, Università Degli Studi di Bari “Al-do Moro”, 70124 Bari, Italy; mariasevera.dicomite@uniba.it; 2 Dipartimento dell’Emergenze e Trapianti di Organi (D.E.T.O.), Sezione di Cliniche Veterinarie e Produzioni Animali, Università Degli Studi di Bari “Aldo Moro”, 70124 Bari, Italy; cosimo.esposito@gmail.com (C.E.); guarracinovet@gmail.com (A.G.); eddagiuseppina.francioso@uniba.it (E.F.); francesco.staffieri@uniba.it (F.S.); antonio.crovace@uniba.it (A.C.)

**Keywords:** UAP, dog, bone marrow mononuclear cells, anconeal process

## Abstract

The aim of this study was to report the results of autologous bone marrow mononuclear cell (BMMC) transplantation as a minimally invasive treatment for grade 2 UAP in dogs. This was an observational case series on six German shepherd dogs affected by grade 2 UAP as defined according to their clinical condition as well as radiographic and CT findings. Bone marrow was collected from the iliac crest and the mononuclear fraction was separated with density gradient centrifugation. Cells were suspended in fibrin glue before BMMC administration and implanted via transcutaneous injection under IB or CT guidance, using a spinal needle directly inserted into the ossification centre between the anconeal process and the olecranon. Clinical and radiographic follow-up was performed for up to 6 months. Microradiographic assessment was performed on one dog that died of other causes. A progressive reduction of pain within 3 weeks after BMMC administration was observed in all dogs, with gradually increased weight bearing on the affected limb. Radiographic and CT follow-up revealed the progressive fusion of the ossification centre at 90 days without any signs of secondary OA. The examination of microradiographs showed newly formed bone tissue in which a residue of calcified cartilage was present at the site of BMMC implantation. On the basis of these results, BMMC therapy for grade 2 UAP may be considered to be an effective and minimally invasive treatment option for dogs.

## 1. Introduction

The anconeal process articulates with the humerus by contacting the supratrochlear foramen at the olecranon fossa, forming the proximal end of the trochlear notch. In most large breed dogs, this mechanism has its own ossification centre and helps to stabilize the elbow joint by restricting mediolateral movement [1]. The anconeal process begins to mineralize between the ages of 10 and 16, with complete fusing to the ulna occurring between the ages of 16 and 20 weeks. In cases where the anconeal process fails to fuse with the ulna, the lesion, called an ununited anconeal process (UAP), is well known in the veterinary literature and referred to as elbow dysplasia in dogs [1,2,3]. Originally described as ectopic sesamoid bone at the canine elbow [4], the lesions have not been proven to be genetically influenced, and other factors that are believed to be associated with UAP are nutrition, hormonal influences, excess weight [3,5,6,7,8,9,10], and trauma, but a logical biomechanical aetiology of a physeal fracture is based on force and moment. This lesion is generally observed in young dogs of large breeds, especially German shepherds, Labradors, and golden retrievers, as well as in achondroplastic breeds, after six months of age. [10] A UAP can be bilateral, resulting in instability, anconeal process displacement, and secondary osteoarthritis of the elbow joint. Osteochondrosis and growing disruption of the proximal ulna elliptical semilunar notch with inadequate articulation with the humerus can be risk factors for the development of UAP, resulting in greater pressure against the anconeal process. [11].

The criteria for evaluating pathological progression are based on X-ray examinations and the intra-surgical condition of the anconeal process. Bardet (1998), and later Vezzoni and Benjamino (2020) determined the degrees of severity by evaluating the fissuration (opaque in grade 1 and transparent in grades 2 and 3) using X-ray examination and the mobility of the anconeal process using intra-surgical evaluation (attached in grade 1 and low mobility in grades 2 and 3). Grades 4 and 5 of severity show an incongruous and dislocated anconeal process with bone reabsorption by X-ray examination and a detached anconeal process by intra-surgical evaluation [12,13] (Table 1).

Proximal ulnar osteotomy was first described by Olsson in 1990 [14]. The theory behind this treatment is that elbow incongruity is caused by a shorter ulna relative to the radius (negative radioulnar incongruence), which puts too much stress on the anconeal process and prevents a proper union. Theoretically, osteotomy allows the triceps to proximally displace the proximal segment, reducing tension on the anconeal process and allowing bone union to occur. Indeed, displacement of the proximal ulnar segment is a more complex three-dimensional tilting that can influence surgical techniques including osteotomy vs. ostectomy, transverse vs. oblique osteotomy, and intramedullary pin fixation vs. no fixation [15,16,17]. In 2007, osteotomy and distraction of ulna by external fixator were also proposed [18]. While the original studies of ulnar osteotomy were very promising, subsequent investigations have failed to attain such high union rates, and a combination of ulnar osteotomy and UAP fixation for severely unstable pieces has been advocated. However, the lag screw fixation technique was also demonstrated to have a high failure rate (failed union and screw breakage) and, therefore, should be considered with caution only in selected cases [19,20]. It is possible that ulnar osteotomy is not necessary in dogs with normal elbow congruence, although this has not been proven. In elderly dogs and/or dogs with substantial elbow osteoarthritis, attempts to achieve bony union of the UAP may not be required, UAP excision is indicated. Recently, many studies on treatment options for avascular necrosis have proposed the use of bone marrow (BM) and BM-derived cells [21,22,23]. These cell-based techniques have been employed in dogs to verify their efficacy as a translational model [24,25,26,27]. Bone marrow mesenchymal stem cells are an effective therapeutic agent for bone regeneration and disorders affecting the epiphyseal discs due to a variety of favourable biological properties [28,29,30].

The purpose of this observational retrospective study is to report the results obtained from autologous bone marrow mononuclear cell (BMMC) implantation for the treatment of grade 2 UAP in six German shepherd dogs as an alternative treatment for UAP in the first (1°, 2°, and 3°) phases of dislocation.

## 2. Materials and Methods

### 2.1. Inclusion Criteria

This case series included the clinical records of German shepherd dogs affected by unilateral UAP and treated with bone marrow stromal cell (BMSC) implantation between 2016 and 2019. German shepherd breed, aged between seven and nine months, unilateral localization of UAP, and lack of obvious degenerative lesions of the elbow joint were the inclusion criteria [31,32]. Dogs having concomitant elbow lesions (fragmented medial coronoid process, osteochondrosis dissecans of the medial humeral condyle, and elbow incongruity) and osteoarthritis signs in either the UAP afflicted or at controlateral elbow were excluded from this study.

### 2.2. Clinical Examination

All the dogs underwent complete clinical orthopaedic examinations that included subjectively observation and gait analysis, as well as bone and articular manipulation. All limbs were examined, and muscle atrophy and soft tissue swelling evaluated. Pain, joint crepitation during passive manoeuvres, and range of motion were evaluated by hyperextension and supination of the elbow.

### 2.3. X-ray and CT Evaluation

Bilateral X-rays of the elbows at 45° and 110° and computed tomography were performed with the dogs under general anaesthesia. Three standard radiographic views were observed, which included the following: extended mediolateral, flexed mediolateral, and craniocaudal.

Computed tomography scans were performed with a CT Pro Speed Scanner (General Electric Medical Systems, Milwaukee, WI, USA). All of the dogs were positioned in a dorsal recumbent position, with both thoracic limbs extended cranially in a symmetrical manner. Using a bone reconstruction technique, contiguous, transverse 1 mm thick slices were obtained from the proximal aspect of the olecranon to 1 cm distal to the elbow joint and parallel with the humeroradial joint region. A bone setting was used to review images. For the examination of UAP features, transverse images of the elbow joint were reformatted in dorsal and sagittal planes.

### 2.4. Bone Marrow Harvesting

The treatment was performed according to the Italian guidelines on the use of stem cells for clinical applications in veterinary medicine (Gazzetta Ufficiale, Italy, no. 277, 26 November 2013), and each owner provided informed consent.

**Figure 1:**
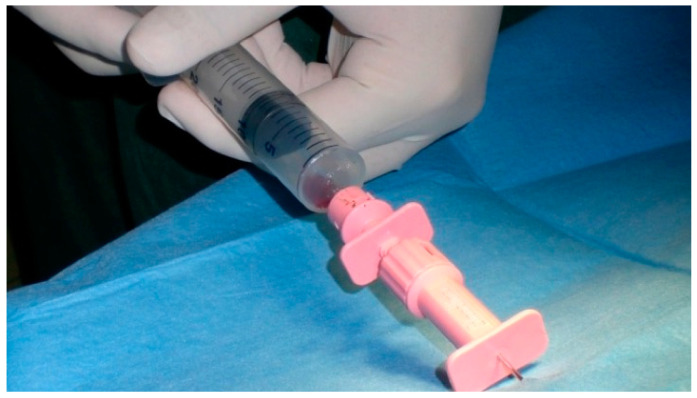
Bone marrow collection from the iliac crest.

After sedation with acepromazine (0.2 mg/kg) (Prequillan, Fatro, Italy) and methadone (0.3 mg/kg) (Semfortan, Dechra, Spain), induction and maintenance of anaesthesia were obtained with propofol (5 mg/kg) (Propovet, Zoetis, Italy). The hip regions were prepared for aseptic surgery. The autologous BMMCs were obtained from bone marrow collected from the iliac crest of each dog by means of a Jamshidi biopsy needle connected to a heparinised syringe (2500 IU/20 mL BM). A minimum volume of 20 mL of bone marrow was collected. If this amount was not reached with a single site, then another harvest was performed from the other iliac crest (Figure 1).

### 2.5. Bone Marrow Processing and BMMC Isolation

The bone marrow was diluted 1:1 in PBS before being stratified 1:1 over Biocoll separating solution (Ficoll, gradient 1.077 g/mL, Biochrom, Leonopenstr 2–6, D-12247, Berlin, Germany) and centrifuged for 30 min at 2000 rpm. Nuclear staining (0.1 percent methyl violet in 0.1 M citric acid) was used to count the separated cells. For the next dilution in an acceptable amount of fibrin glue, the cells were washed twice with phosphate buffer solution (PBS) (Tissucol, Baxter Spa, Rome, Italy).To test the cells’ ability to form colony-forming unit fibroblasts (CFU-F), 100 L of cells were seeded in two 100 mm diameter Petri dishes containing complete medium (Coon’s F-12 medium, 10% FBS, 100 IU/mL penicillin and streptomycin, and 5% of a 200 M solution of L-glutamine (Biochrom, Leonopenstr 2–6, D-1), before and after density gradient centrifugation. The cells were then rinsed in PBS with a pH of 7.2, fixed in 4% buffered formalin, and stained with 1% methylene blue in borate buffer (10 mM, pH 8.8).

### 2.6. BMMC Injection

**Figure 2:**
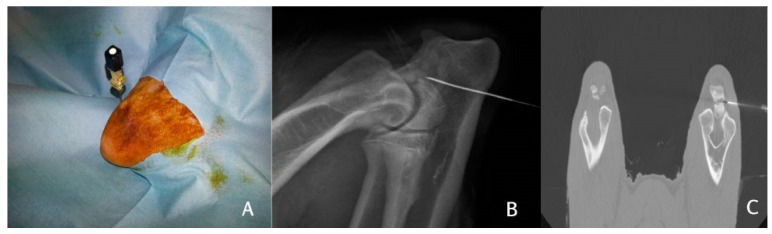
Injection of the stem cells into the ossification centre. The spinal needle is inserted into the anconeal process (**A**) and an X-ray (**B**) or a CT (**C**) is performed to verify the position of the needle. The stem cells are injected after correct positioning is verified.

One hour after the harvesting of bone marrow, the dogs were re-anaesthetised with propofol (Propovet, Zoetis, Rome, Italy) (5 mg/kg) and the anaesthesia maintained with isoflurane (Iso-vet, Piramal Clinical Care Spa, Italy) and oxygen. In three of the dogs (*n* = 3), BMMCs were administered under C-arm fluoroscopy, whereas in three other dogs (*n* = 3), BMMCs were administered under CT-guided injection. The BMMCs were suspended in 1 mL of fibrinogen and injected using a 22-gauge spinal needle directly inserted into the ossification centre between the anconeal process and the olecranon (Figure 2).

Immediately after the injection of BMMCs + fibrinogen, 0.3 mL of thrombin was injected through the same needle to obtain a clot of cells and fibrin glue in the injection site. At the end of the procedure, all dogs were recovered from general anaesthesia.

### 2.7. Post-Surgical Regimen

In the post-operative period, amoxicillin/clavulanic acid (oral administration, 25 mg/kg every 12 h) and tramadol (4 mg/kg every 12 h) were administered for 7 days. NSAIDs (Non-steroidal anti-inflammatory drugs) were avoided to exclude any interference with BMMC effects. The absence of pain was evaluated during post-surgery controls performing elbow joints movements and reporting, while the dog was walking, the presence of lameness. An orthopaedic examination, X-rays, and computed tomography were performed at 60, 90, 180, and 270 days after the treatment.

### 2.8. Complications

Major complications included haemorrhage, fracture of the iliac crest, and infection at the harvest and administration sites. Minor complications such as inflammation and pain or discomfort at the donor site or in the joint were considered.

## 3. Results

The clinical records of 47 dogs were reviewed, and six German shepherd dogs who matched the inclusion criteria were treated with bone marrow mononuclear cells, namely three males and three females with a mean body weight of 29 ± 2.3 kg and a mean age of 7.3 ± 0.5 months. All dogs were referred for confirmation of monolater lameness, which was an inclusion criterion. At orthopaedic examination, all animals showed external rotation of the paw of the affected limb at seated and standing posture. During walking, the dogs showed a reduction in weight bearing of the affected limb with grade 1 or 2 lameness. In all cases, muscle atrophy of the shoulder and a moderate swelling between the lateral humeral epicondyle and the olecranon were present. At passive movements of the elbow, a 5% to 10% reduction in the range of motion and pain signs at hyperextension and supination were present in all cases. Crepitation was absent in all cases.

### 3.1. X-ray Results

In all dogs, the mediolateral flexed view of the elbows showed the presence of grade 2 UAP without any signs of secondary osteoarthritis. The radiographic examination of the contralateral joint revealed, at the same time, the complete union of the ossification centre of the anconeal process.

### 3.2. CT Results

The computed tomography study revealed evident and irregular fragmentation of the anconeal processes with a complete radiolucent line separating the anconeal process from the ulna. The CT images were reviewed using a bone setting of transverse images of the elbow joint in dorsal and sagittal planes and confirmed the lesion was associated with a moderate grade of elbow incongruence. Signs of other elbow abnormalities were not evident in the dogs included in the study. Although all the dogs underwent controls at 180 days, one of them missed the 270 days follow up since the owner did not agree to the dog undergoing further investigations.

### 3.3. Bone Marrow Results

A mean of 34.33 ± 5.96 mL of bone marrow were harvested. The number of BMMCs obtained after Ficoll separation was 2.57 × 10^7^ ± 1.30 × 10^7^ cells. The total CFU/mL of bone marrow was 528.5 ± 224.3 CFU/mL (Table 2).

Harvesting from a single iliac crest was performed in all dogs. No major complications or infections were seen after bone marrow harvesting or after intraosseous injection of BMMCs into the lesion.

### 3.4. Follow-Up

**Figure 3:**
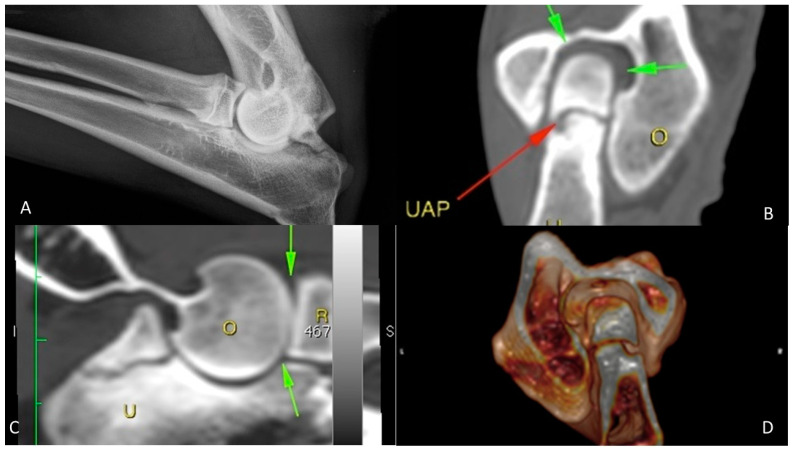

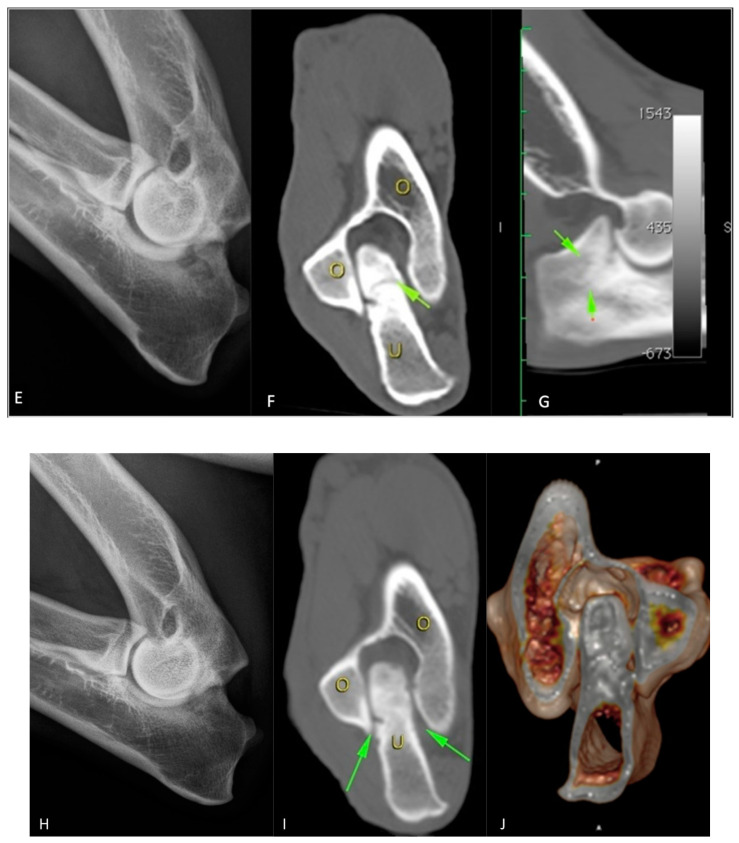
German shepherd female, 7 months old; grade 2 UAP. (**A**–**D**) Preoperative X-ray and CT exams; (**E**–**G**) post-operative control at 60 days; (**H**–**J**) post-operative control at 270 days. The arrows underline the anconeal process reparation. The O stands for humerus and the U stands for ulna.

The first control was performed at three weeks after treatment: dogs did not show any local or systemic adverse reactions, such as infections, swelling or leaking from the injection site. The clinical examination showed a progressive reduction in pain within 3 weeks after BMMC administration upon gradually increased weight bearing on the affected limb. The carpus valgus attitude was still appreciated. A progressive reabsorption of synovial fluid resulted in a reduction in joint effusion to a complete remission of clinical signs at 3 months post stem cell injection. The radiographic and computed tomography examinations, performed at 21, 90, 180, and 270 days after the treatment, revealed progressive fusion of the ossification centre, which was completed at 90 days without any signs of secondary OA after 270 days follow-up (Figure 3).

### 3.5. Microradiographic Assessment

For one dog that died of unrelated causes (complications of patent ductus arteriosus) at 64 days after treatment, its bone tissue was harvested on which microradiographic assessment was conducted. The bone was fixed in 4% paraformaldehyde in 0.01 M phosphate-buffered saline solution (PBS pH 7.2) for 2 days at 4 °C, thoroughly rinsed in running water for 2 h, dehydrated in ethanol, and conventionally processed for methylmethacrylate embedding. Serial sections of 100 µm thickness were obtained according to the coronal plane by means of a circular diamond-bladed saw (SP1600, Leica Microsystems, Wetzlar, Germany), polished under running water, and air dried in an incubator at 37 °C for 24 h. Sections were coded and microradiographed at 8 kV and 14 mA using an XRG-3000 X-ray generator (Ital Structures Research, Riva Del Garda, Italy) and Kodak high resolution film. Contact microradiographs were developed in Kodak HC-110 solution, fixed in Ilford Hypam rapid fixer (HARMAN technology Ltd, Ilford Way, Mobberley, Cheshire, UK), washed in double distilled water, and air dried at room temperature. The histological analysis, using a light microscope (Nikon Eclipse E 400, Tokyo, Japan), linked with a digital camera (Nikon DS-5M) and image processing software (Nikon Nis-Elements BR), was carried out on the microradiographs. In all the described cases, grade 2 UAP, according to IEWG (International Elbow Working Group), was also confirmed by CT examination.

**Figure 4:**
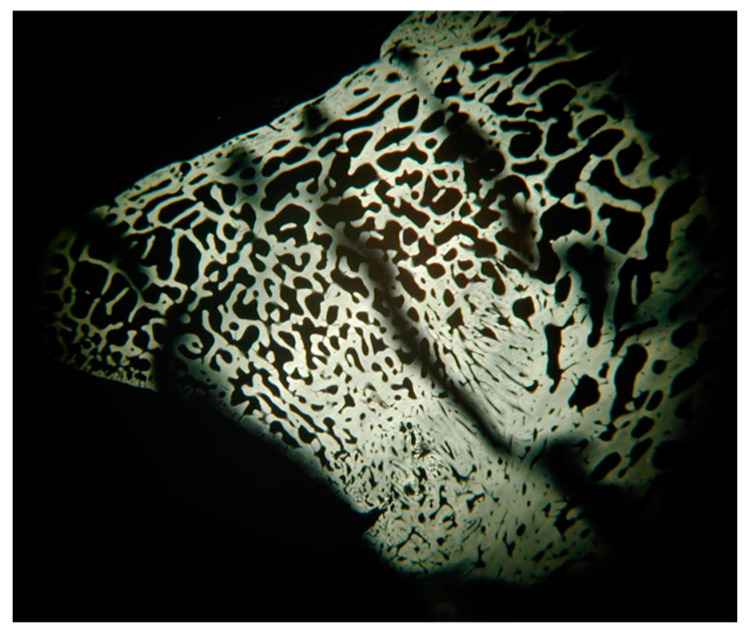
Microradiograph of bone tissue taken from the anconeal process.

The healing area appeared to be slightly radiolucent as compared with the surrounding bone. In Figure 4, it is possible to observe the presence of newly formed bone tissue (blue arrow) crossed by numerous vascular channels of variable amplitude and irregular course which often flow together. Within this area, a calcified cartilage residue invaded by vascular structures is still recognisable. It appears to be more radiopaque and provides evidence of secondary healing consisting of endochondral ossification. Bypassing this region, more internally along the conjunction area, the bone tissue appears to be more regular, and the arrangement of the vascular channels follows a radial course (Figure 4).

## 4. Discussion

The results obtained in this case series showed that the use of BMMCs, as a minimally invasive treatment for grade 2 UAP in dogs, is feasible and effective producing prompt ossification, rapid recovery from pain, and also leading to gradual increase in weight bearing of the affected limb, up to a complete remission of clinical signs at 3 months after stem cell injection, confirming same recent studies on the use of stem cell in animals.

In dogs affected by UAP, the best chance of achieving a good outcome is by reattaching the ununited anconeal process. The techniques currently used have different results depending on the presence of many variables such as age of dogs, severity of injury, and presence of incongruent joint and osteoarthritis. In the case of minimal clinical signs, a conservative treatment is suggested, consisting of weight management, exercise modification, and use of NSAIDs to control pain, but this therapy does not significantly alter the progression of osteoarthritis, and comfortable use of the limb is not achieved [20]. The various surgical techniques described for treatment of UAP do not always give good results in terms of functional recovery and their effects depend on the grade of the lesions, the time in which the surgical therapy is implemented, and the grade of joint impairment. Simple fragment removal often results in significant short-term improvement in patient comfort and function, although progressive osteoarthritis and associated morbidity are the predictable long-term sequelae.

Although the clinical efficacy of BMMC therapy for bone lesion repair has been demonstrated in human medicine, the function of transplanted cells is unknown. The immunohistochemical findings in a recent study [33] on the usage of BMMCs revealed an increase in bone thickening with substantial neoangiogenesis and the presence of CD34+ cells (a marker of BMMCs). The regenerative effect could have been caused by cell therapy, either directly or indirectly via cell attraction and chemotaxis, as shown in other mesenchymal tissues [34]. Our findings demonstrated clinical remission of symptoms within 3 weeks after treatment with a clear improvement in terms of pain reduction as shown by the remission of lameness and also underline the ossification of the anconeal process. BMMCs release a wide range of factors that have immunomodulatory, anti-inflammatory, anti-apoptotic, proangiogenic, proliferative, or chemo-attractive properties [20,34,35,36,37,38,39,40]. The ability of stromal cells to reduce pain is most likely related to their anti-inflammatory properties. Indeed, mesenchymal stromal cells (MSCs) have regenerative and anti-inflammatory potential [41,42,43], and bone marrow-derived MSCs have been shown to inhibit T-cell proliferation [26]. Currently, the immunomodulatory characteristics of BMSCs are attributed to a number of soluble factors that are either produced constitutively by BMSCs or as a result of interaction with target immune cells [28,33,34]. Moreover, the radiographic evaluation showed appreciable healing of the bone defect at 90 days after treatment without significant signs of OA up to 270 days. Some authors have suggested that the efficiency of the BMSCs could be related to the availability of stem cells endowed with osteogenic properties in the lesion, which increases after bone marrow implantation [35,43]. Another possible explanation for the effects of bone marrow implantation is that the injected BMSCs secrete angiogenic cytokines, resulting in increased angiogenesis and subsequent improvement in osteogenesis [28]. Jiang et al. demonstrated that in vitro cultured cells were not suitable for cell therapies and that fresh BMSCs were the best choice [36].

The single case in which the microradiographic assessment was performed demonstrated a clear osteoinductive effect of the treatment with endochondral ossification and regular bone formation.

In our study, no dogs observed complications related to the BM harvest or at the administration site, in line with other animal studies generally it is considered to be a safe procedure [37]. However, minor and major complication were reported in human beings. Indeed, a recent systematic review, [37] the authors stated that possible complication reported are usually related to the harvest or during BMMNC (Bone Marrow Mononuclear Cells) application. In particular, it has been reported breaches of site of harvest (iliac crest) in obese patients, injury to external iliac artery associated with sciatic nerve and gluteal vessel injury [38]. In another study it has been reported a haemorrhage rate of 0.0005% [39]. Infections at the harvest site was rarely reported and successfully treated with antibiotics. Chronic pain was also reported but related to the site of harvest and spontaneously resolved [40].

In the administration site infection is a potential risk, but antibiotic prophylaxis and standard clean surgical practice reduced significantly the risk [41].

In dogs it has been reported a fatal fat embolism to lungs [42]. However, it has been reported exclusively, for intra-osseous administration [42].

The limitations of this study are strictly associated with the low number of dogs that fit the inclusion criteria and could be treated with stem cells. In our study, we included cases that were grade 2 according to the UAP scale by Vezzoni. Moreover, the inclusion criteria limited the study group to monolateral cases without clear degenerative lesions and with the absence of associated lesions. The other limitation is linked to the semi-quantitative evaluation of the clinical outcomes since is based on the evaluations on lameness. An important evaluation may require pressure plates to evaluate in a more objective manner the load bearing of the animals before and after the treatment.

Another issue related to the study is the absence of a control group that could corroborate the efficiency of the treatment and it should be taken in consideration for future studies.

The retrospective design of this study also limited the case selection. A further prospective randomised control study should be conducted to strengthen our current results. Furthermore, the number of BMMCs obtained from the bone marrow had to be at least 2.3 × 10^7^ to obtain good regeneration in treatment of the lesion, since the suggestion in the current literature is to use the largest number of cells available [43]. In addition, the maximum follow-up was 270 days post-treatment and, therefore, it was not possible to evaluate the long-term progression of osteoarthritis. Finally, it is important to highlight that it was not possible to perform histological examinations on the treated dogs since all were privately owned and not accessible.

## 5. Conclusions

The results obtained in these cases, if confirmed from other studies, indicate that in the case of early diagnosis of the disease, cell therapy could be a possible alternative to traditional surgical treatment for UAP in dogs.

## Figures and Tables

**Table 1:** Classification of UAP based on Vezzoni and Benjamino Scale.

	X-ray	Surgery
Grade 1 (1°)	Opaque fissure	Completely attached anconeal process
Grades 2 and 3 (2–3°)	Transparent fissure	Low mobility anconeal process
Grades 4 and 5 (4–5°)	Incongruous anconeal process with dislocation and bone reabsorption	Detached anconeal process

**Table 2:** BMMCs quantity from the harvesting of bone marrow.

Variable	Mean	St Dev	Median	Interquantile Range
Age (months)	7.7	0.8	7.5	2.0
Bone marrow volume (ml)	34.33	5.96	35.00	16.00
No. BMMCs	2.57 × 10^7^	1.30 × 10^7^	2.30 × 10^7^	3.70 × 10^7^
UFC/ml	528.5	224.3	495	500

## Data Availability

Data is contained within the article.

## References

[1] Stiern R.A. (1956). Ectopic sesamoid bones at the elbow (*Patella cubiti*) of the dog. J. Am. Veter-Med. Assoc..

[2] Lenehan T.M., Van Sickle D.C. (1985). Ununited Anconeal Process, Ununited Coroneal Process, Ununited Medial Epicondyle, Patella Cubiti and Sesamoidale Fragments of the Elbow.

[3] Corley E.A., Sutherland T.M., Carlson W.D. (1968). Genetic aspects of canine elbow dysplasia. J. Am. Veter-Med. Assoc..

[4] Harasen G. (2009). Orthopedics: Ununited anconeal process. Can. Vet. J..

[5] Swenson L., Audell L., Hedhammar A. (1997). Prevalence and inheritance of and selection for elbow arthrosis in Bernese mountain dogs and Rottweilers in Sweden and benefit: Cost analysis of a screening and control program. J. Am. Veter-Med. Assoc..

[6] Olsson S.E. Osteochondrosis of the canine elbow joint. Pathogenesis and a new approach to the surgical treatment of UAP. Proceedings of the 18th Congress-European Society of Veterinary Surgery.

[7] Hazewinkel H.A.W. (1989). Nutrition in relation to skeletal growth deformities. J. Small Anim. Pr..

[8] Hazewinkel H.A.W. (1993). Nutrition in Orthopedics. Disease Mechanisms in Small Animal Surgery.

[9] Grøndalen J., Lingaas F. (1988). Arthrosis of the elbow joint among Rottweiler dogs. Results from investigations into hereditary disposition. Tijdschr. Voor Diergeneeskd..

[10] Wind A.P., Packard M.E. (1986). Elbow incongruity and development elbow disease in the dog: Part I. J. Am. Hosp. Assoc..

[11] Sjöström L., Kasström H., Källberg M. (1995). Ununited anconeal process in the dog. Pathogenesis and treatment by osteotomy of the ulna. Vet. Comp. Orthop. Traumatol..

[12] Vezzoni A., Benjamino K. (2021). Canine Elbow Dysplasia. Veter-Clin. North Am. Small Anim. Pract..

[13] Bardet J. Classification and treatment of ununited anconeal process in dogs. Proceedings of the 9th ESVOT Congress.

[14] Turner B.M., Abercromby R.H., Innes J., McKee W.M., Ness M.G. (1998). Dynamic Proximal Ulnar Osteotomy for the Treatment of Ununited Anconeal Process in 17 Dogs. Veter-Comp. Orthop. Traumatol..

[15] Ferrigno C.R., Schmaedecke A., Sterman F.A., Lincoln J. (2007). Treatment of ununited anconeal process in 8 dogs by osteotomy and dynamic distraction of the proximal part of the ulna. Pesqui. Veterinária Bras..

[16] Fox S.M., Burbidge H.M., Bray J.C., Guerin S.R. (1996). Ununited anconeal process: Lag-screw fixation. J. Am. Anim. Hosp. Assoc..

[17] Pettitt R.A., Tattersall J., Gemmill T., Butterworth S.J., O’Neill T.J., Langley-Hobbs S.J., Comerford E.J., Innes J.F. (2009). Effect of surgical technique on radiographic fusion of the anconeus in the treatment of ununited anconeal process. J. Small Anim. Pract..

[18] Hulse D.A., Bähr A., Jerram R.M., Krotscheck U. (2000). Ununited anconeal process: Lag-screw fixation with proximal ulnar osteotomy. Veter-Comp. Orthop. Traumatol..

[19] Gangji V., Hauzeur J.-P., Matos C., De Maertelaer V., Toungouz M., Lambermont M. (2004). Treatment of Osteonecrosis of the Femoral Head with Implantation of Autologous Bone-Marrow Cells. JBJS.

[20] Körbling M., Estrov Z. (2003). Adult Stem Cells for Tissue Repair—A New Therapeutic Concept?. N. Engl. J. Med..

[21] Cabrolier J., Molina M. (2016). Is instillation of bone marrow stem cells at the time of core decompression useful for osteonecrosis of the femoral head?. Medwave.

[22] Jin H., Xia B., Yu N., He B., Shen Y., Xiao L., Tong P. (2012). The effects of autologous bone marrow mesenchymal stem cell arterial perfusion on vascular repair and angiogenesis in osteonecrosis of the femoral head in dogs. Int. Orthop..

[23] Wang B.-L., Sun W., Shi Z.-C., Zhang N.-F., Yue D.-B., Guo W.-S., Xu S.-Q., Lou J.-N., Li Z.-R. (2009). Treatment of nontraumatic osteonecrosis of the femoral head with the implantation of core decompression and concentrated autologous bone marrow containing mononuclear cells. Arch. Orthop. Trauma Surg..

[24] Crovace A., Favia A., Lacitignola L., Di Comite M.S., Staffieri F., Francioso E. (2008). Use of autologous bone marrow mononuclear cells and cultured bone marrow stromal cells in dogs with orthopaedic lesions. Veter-Res. Commun..

[25] Crovace A., Lacitignola L., Rossi G., Francioso E. (2010). Histological and Immunohistochemical Evaluation of Autologous Cultured Bone Marrow Mesenchymal Stem Cells and Bone Marrow Mononucleated Cells in Collagenase-Induced Tendinitis of Equine Superficial Digital Flexor Tendon. Veter-Med. Int..

[26] Luzzi S., Crovace A.M., Del Maestro M., Lucifero A.G., Elbabaa S.K., Cinque B., Palumbo P., Lombardi F., Cimini A., Cifone M.G. (2019). The cell-based approach in neurosurgery: Ongoing trends and future perspectives. Heliyon.

[27] Sabino L., Maria C., Luca L., Valerio V., Edda F., Giacomo R., Gloria I., Juan G., Antonio C. (2018). Engraftment, neuroglial transdifferentiation and behavioral recovery after complete spinal cord transection in rats. Surg. Neurol. Int..

[28] Crovace A.M., Lacitignola L., Staffieri F., Francioso E., Rossi G., Crovace A. (2020). Treatment of Monolateral Legg-Calvé-Perthes Disease with Autologous Bone Marrow Mononuclear Cells in 32 Dogs. VCOT Open.

[29] Hazewinkel H.A.W. Screening for Elbow Dysplasia, grading according to the IEWG. Proceedings of the 30th Annual Meeting IEWG.

[30] Ohlerth S., Tellhelm B., Amort K., Ondreka N. Explanation of the IEWG grading system. Proceedings of the 30th Annual Meeting IEWG.

[31] Samoy Y., Gielen I., van Bree H., Van Ryssen B. (2011). Dysplastic elbow diseases in dogs. Vlaams Diergeneeskd. Tijdschr..

[32] Wind A.P., Packard M. (1986). Elbow incongruity and developmental elbow diseases in the dog: Part II. J. Am. Anim. Hosp. Assoc..

[33] Wang D., Zhang H., Liang J., Li X., Feng X., Wang H., Hua B., Liu B., Lu L., Gilkeson G.S. (2013). Allogeneic Mesenchymal Stem Cell Transplantation in Severe and Refractory Systemic Lupus Erythematosus: 4 Years of Experience. Cell Transplant..

[34] Wang M., Liao Q., Zhou B., Qiu Z.-Q., Cheng L.-M. (2013). Preliminary study of influence of bone tissue from osteonecrosis of femoral head on the proliferation and differentiation of canine bone marrow mesenchymal stem cells. Zhonghua Yi Xue Za Zhi.

[35] Murphy M.B., Moncivais K., Caplan A. (2013). Mesenchymal stem cells: Environmentally responsive therapeutics for regenerative medicine. Exp. Mol. Med..

[36] Jiang T., Xu G., Wang Q., Yang L., Zheng L., Zhao J., Zhang X. (2019). Correction: In vitro expansion impaired the stemness of early passage mesenchymal stem cells for treatment of cartilage defects. Cell Death Dis..

[37] Imam M.A., Holton J., Ernstbrunner L., Pepke W., Grubhofer F., Narvani A., Snow M. (2017). A systematic review of the clinical applications and complications of bone marrow aspirate concentrate in management of bone defects and nonunions. Int. Orthop..

[38] Hernigou J., Picard L., Alves A., Silvera J., Homma Y., Hernigou P. (2014). Understanding bone safety zones during bone marrow aspiration from the iliac crest: The sector rule. Int. Orthop..

[39] Bain B.J. (2003). Bone marrow biopsy morbidity and mortality. Br. J. Haematol..

[40] Burkle C.M., Harrison B.A., Koenig L.F., Decker P.A., Warner D.O., Gastineau D.A. (2004). Morbidity and mortality of deep sedation in outpatient bone marrow biopsy. Am. J. Hematol..

[41] Husebye E.E., Lyberg T., Røise O. (2006). Bone marrow fat in the circulation: Clinical entities and pathophysiological mechanisms. Injury.

[42] Orlowski J.P., Julius C.J., E Petras R., Porembka D.T., Gallagher J.M. (1989). The safety of intraosseous infusions: Risks of fat and bone marrow emboli to the lungs. Ann. Emerg. Med..

[43] Hernigou P., Beaujean F., Lambotte J.C. (1999). Decrease in the mesenchymal stem-cell pool in the proximal femur in corticosteroid-induced osteonecrosis. J. Bone Jt. Surgery. Br. Vol..

